# A review on lymphocyte radiosensitivity and its impact on radiotherapy

**DOI:** 10.3389/fonc.2023.1201500

**Published:** 2023-08-03

**Authors:** Harald Paganetti

**Affiliations:** ^1^ Department of Radiation Oncology, Massachusetts General Hospital, Boston MA, United States; ^2^ Harvard Medical School, Boston MA, United States

**Keywords:** lymphopenia, lymphocytes, radiotherapy, radiosensitivity, blood dose

## Abstract

It is well known that radiation therapy causes lymphopenia in patients and that this is correlated with a negative outcome. The mechanism is not well understood because radiation can have both immunostimulatory and immunosuppressive effects. How tumor dose conformation, dose fractionation, and selective lymph node irradiation in radiation therapy does affect lymphopenia and immune response is an active area of research. In addition, understanding the impact of radiation on the immune system is important for the design and interpretation of clinical trials combining radiation with immune checkpoint inhibitors, both in terms of radiation dose and treatment schedules. Although only a few percent of the total lymphocyte population are circulating, it has been speculated that their increased radiosensitivity may contribute to, or even be the primary cause of, lymphopenia. This review summarizes published data on lymphocyte radiosensitivity based on human, small animal, and *in vitro* studies. The data indicate differences in radiosensitivity among lymphocyte subpopulations that affect their relative contribution and thus the dynamics of the immune response. In general, B cells appear to be more radiosensitive than T cells and NK cells appear to be the most resistant. However, the reported dose-response data suggest that in the context of lymphopenia in patients, aspects other than cell death must also be considered. Not only absolute lymphocyte counts, but also lymphocyte diversity and activity are likely to be affected by radiation. Taken together, the reviewed data suggest that it is unlikely that radiation-induced cell death in lymphocytes is the sole factor in radiation-induced lymphopenia.

## 1 Introduction

Radiation-induced lymphopenia (RIL) has long been observed in radiation therapy patients (1–3) and develops in up to ~70% of patients undergoing external beam radiation therapy (4–8). High-grade RIL has been shown to correlate with poor overall survival, disease recurrence, and metastasis rates (9). A correlation between lymphopenia and dose to circulating lymphocytes has been demonstrated (e.g., (6, 8, 10–13). Therefore, it has been speculated that lymphopenia is caused by an increased radiosensitivity of circulating lymphocytes (7) and the large volume of blood irradiated during radiotherapy.

Treatment delivery techniques differ in the distribution of the low dose bath outside of the planned treatment volume and in the duration of treatment in a fraction (10, 14, 15) resulting in different dose distributions experienced by circulating lymphocytes (6, 16–19). In a study of esophageal cancer, 35% of patients had grade 4 RIL when treated with concurrent chemotherapy and either intensity-modulated photon (IMRT) or proton therapy, which was correlated with overall survival (20). Due to the lower integral dose, patients treated with protons had 70% less grade 4 RIL compared to IMRT. However, this was not confirmed in a study of 150 oropharyngeal cancer patients (21) and in locally advanced non-small cell lung cancer (NSCLC) treated with either IMRT or proton therapy (15). Dose to lymphocytes is also influenced by patient specific factors such as baseline levels of absolute counts and lymphocyte subpopulations, which are known to differ between patient groups (22), as well as fractionation and dose rate (18, 23–27). Consequently, lymphocyte sparing radiation therapy has been proposed (8, 28). Smaller target volumes and hypo-fractionated regimens may be associated with higher post-treatment lymphocyte counts. For example, during a 30-fraction treatment with 2 Gy/fraction to a target volume of 8 cm in diameter, 95% of the circulating blood receives doses greater than 0.5 Gy, with a mean dose to the circulating blood greater than 2 Gy (8). Larger field sizes increased chromosomal aberrations in circulating lymphocytes in a prospective series of lung cancer patients treated with carbon-ion therapy (29) and were associated with lower post-treatment lymphocyte counts in lung cancer treated with protons (15). There have been several other studies of field size effects on lymphopenia in solid tumors (5, 30, 31).

Although the amount of circulating blood plays a role, considering that only a few percent of the total lymphocyte population is circulating, compared to those residing in organs or lymph nodes, is not clear whether RIL is simply caused by radiation-induced depletion of circulating lymphocytes. Radiation has deleterious effects not only on circulating lymphocytes but also on tumor-infiltrating lymphocytes and lymphocytes residing in structures such as the bone marrow (32), spleen (33), and lymph nodes (34). Lymphopenia has been shown to correlate strongly with dose to the spleen (33, 35–37). The capillaries in the spleen are permeable, resulting in high transit times for lymphocytes in the spleen, which in turn results in significant dose to lymphocytes in treatments involving the spleen. By assessing chromosome aberrations in lymphocytes in breast cancer patients, it has been shown that the number of lymph nodes in the field plays a significant role (34). A correlation with bone marrow dose has also been shown by several investigators (32, 38–41), but not by Saito et al. (36). Lymphopenia has also been associated with lymph node irradiation in prostate radiation therapy (42) and breast radiation therapy (43). Consequently, dose constraints to lymphoid organs have been proposed to mitigate lymphopenia (44). Reduced total counts as well as counts in lymphocyte sub-populations were reported for colorectal cancer patients (45) and liver SBRT patients (46).

The interaction of radiation with the immune system is complex (47). Radiation therapy can have both immune-stimulatory (18, 48–52) and immune-suppressive (5) effects. Radiation can promote the release of damage-associated molecular patterns (DAMPs) and tumor antigens via immunogenic cell death, activate the production of type I interferon (IFN) and IFN-stimulated genes via DNA damage that is sensed via the cGAS/STING pathway, and activate antigen-presenting cells, including dendritic cells (DCs) and macrophages (53). Antigen-presenting cells travel through lymphatic vessels to the draining lymph nodes (for instance) where they present antigens to naïve lymphocytes initiating their differentiation into effector and memory cells. Activated lymphocytes returnvia the blood to the tumor site where they recognize tumor antigens and carry out various effector functions. Radiation can also suppress the immune response via IFN-mediated upregulation of immune checkpoint molecules (e.g., PD-L1) (54) and by inducing immune-suppressive populations including myeloid-derived suppressor cells. Additionally, radiation can also directly kill immune cells and thereby modulate the immune response.

While some patients respond favorably to immunotherapy, many develop progressive disease (55). This has led to interest in combining immunotherapy with radiation (56–62). Synergistic combinations of radiation and immunotherapy have shown promise (62–66) as they help to overcome the immunosuppressive tumor microenvironment and thus enhance the therapeutic effect of radiation (67, 68). The optimal sequencing of radiation with immunotherapy (18, 69–72) as well as the best radiation modality for combination therapies (6, 16, 17, 73–76) are being studied extensively. It has even been suggested that low dose whole-body irradiation may improve outcome after subsequent treatment regimens due to radiation induced antigen release (52). Furthermore, pre-clinical data suggest that nodal irradiation may attenuate the combinatorial efficacy of immunotherapy-radiation combination regimens (77). There are numerous clinical trials combining radiation with immunotherapy (78).

While radiation-induced cell death is not the only key parameter when optimizing radiation treatments in this context, it certainly has a profound impact. Section 2 summarizes the published methods for estimating the dose delivered to circulating lymphocytes during radiation therapy. In section 3, studies assessing the radiation sensitivity of lymphocytes are reviewed.

## 2 Estimating the dose to the blood and to circulating lymphocytes in radiation therapy

Under the assumption that the dose to circulating blood is a surrogate for the dose to circulating lymphocytes, several efforts have been made to estimate the blood dose from radiation exposure. To estimate the cumulative blood dose from whole-body irradiation, Molloy et al. developed a blood perfusion model in which the circulation was modeled in a sinusoidal motion between the upper and lower body without regard to individual organs (79). The blood volume was divided into discrete voxels and a statistical dispersion was introduced to reflect the inhomogeneous blood flow in the body. The treatment beam was simulated assuming a time-dependent dose cloud depending on the field size and machine motion.

Yovino et al. (7) calculated the dose to circulating blood for a high-grade glioma patient as a function of dose rate and photon treatment technique. The model uses the three-dimensional dose distributions in the brain and calculates the dose to the blood passing through the radiation field by assuming that 16% of the cardiac output enters the brain with a total blood volume of 5 l and a blood flow velocity of 10 mm/sec. The model includes several simplifications, such as uniformly distributed blood flow without whole-body blood flow dynamics. Another assumption is that blood does not re-enter the treatment field during the duration of a single beam and/or segment. Between beams and between treatment fractions, the cumulative dose was calculated by convolution of the blood dose histograms. The simulations predicted that a single fraction of radiation would deliver 0.5 Gy to 5% of the circulating cells. After 30 fractions, 99% of the circulating blood had received ≥0.5 Gy. Target volume and field size were the most important parameters. This model was also used by Wild et al. (8) who came to similar conclusions.

Basler et al. (80) used dose-volume histograms for liver treatments to estimate the dose to circulating lymphocytes in VMAT. A mean hepatic blood flow velocity of 10 mm/s with a total body blood volume of 5 l was considered. Cardiac output was set at 5 l/min with a circulation time of 60 s for the total blood volume. The model assumes that regional hepatic blood flow is comparable in the different liver segments. Full blood mixing in between fields or fractions was considered and the probability of re-entering a specific liver segment and treatment field was calculated based on the cardiac output and relative volumes of the liver segments. The results show that the dose to the circulating lymphocytes was mainly influenced by the beam-on time and the target volume.

Jin et al. (81) used a similar approach as Yovino et al. to calculate the dose to the blood using a blood flow network consisting of the lungs, heart, large vessels, and body mass. The blood dose and blood volume contributing to each of these compartments during a single fraction were estimated and converted to an equivalent uniform dose, with the total effective blood dose being the sum of the contributions from all irradiated organs. The model was applied to lung treatments, taking into account mean lung dose, mean heart dose, and the integral dose. Blood dose was correlated with radiation-induced lymphopenia. This model was subsequently applied in other studies that demonstrated a correlation between blood dose and lymphopenia in non-small cell lung cancer (82), esophageal cancer (83, 84), and breast cancer (11), especially when the blood dose was above 4 Gy (84).

The dose to the blood was also estimated to analyze the transcriptional response of genes over time in blood samples after irradiation *in vivo* (85, 86). Considering that most of the blood is irradiated during a 2-min treatment time, the authors determined the mean blood dose as a function of the mean dose to the irradiated volume, the irradiated blood volume, and the body blood volume.

Shin et al. developed a compartmental model that considers blood flow throughout the human body based on compartments defined by the ICRP (International Commission on Radiological Protection) (23). The algorithm assumes a dynamic model describing the spatio-temporal distribution of blood particles (BPs) in organs throughout the body using a discrete-time Markov process. Blood transit times were modeled using ICRP reference mean transit time distributions assuming a Weibull distribution. This was then convolved with the time-dependent radiation field delivery. The simulations revealed different dose levels to the circulating blood for brain irradiation compared to liver irradiation even for similar field sizes due to the different blood flow characteristics of the two organs. The authors also showed that the blood dose-volume histogram is highly sensitive to changes in the treatment time, indicating that dynamic modeling of blood flow and radiation delivery is necessary to evaluate dose to the circulating blood.

To add another level of complexity and accuracy, blood dose algorithms have been developed that explicitly consider venous and arterial vascular trees to account for inhomogeneous organ dose distributions and blood flow dynamics. Hammi et al. (87) developed an intracranial blood flow model based on the major cerebral vasculature extracted from patient MRI data and extended with a network of generic brain vessels. The brain model contains more than 1000 vascular pathways. To determine the dose to the circulating blood, Monte Carlo simulations track the propagation of each individual blood particle through the brain and the time-dependent radiation field delivery. The mean dose to the blood pool was estimated after fractions of proton and photon therapy and showed that the fraction of blood volume receiving any dose after the first fraction was significantly lower for proton therapy. Higher dose rates effectively reduced the fraction of blood irradiated to low doses but increased the amount of blood receiving high doses. The model was also applied by Qian et al. (13), who showed that the treatment dose to the whole body, bone, and large blood vessels as well as the modeled dose to circulating lymphocytes were correlated with lymphopenia.

The internal vasculature of the adult liver, including hepatic arterial, hepatic venous, and hepatic portal venous vessel trees, was created within individual lobes of the ICRP adult female and male livers by Correa-Alfonso et al. (88). For each iteration of the algorithm, pressure, blood flow, and vessel radii within each tree were updated as each new vessel was created and connected to a viable bifurcation site. Liver models were created with virtual vasculature of ~6000 non-intersecting straight cylinders representing the circulations of the vascular tree. To combine the vascular trees with a dynamic dose delivery model, the trees were translated into centerlines that can be deformed to account for patient specific organ contours and for BPs entering the liver. An explicit simulation was implemented to track BPs along different vascular pathways through the liver (24). The dosimetric impact of treatment modality, delivery time, and fractionation on circulating blood cells was quantified showing that doses are highly sensitive to the beam-on time and demonstrating the trade-off between low dose to a large fraction of blood cells and high dose to a small fraction of blood cells. It was concluded that proton treatments are not necessarily advantageous in terms of dose to the blood even though they are associated with a lower integral dose because of the importance of the beam-on time. Similar vascular tree models have been developed for the brain (89) and lung (90). Such organ-specific vasculature models can be combined with a Markov chain approach to link them to whole body blood flow based on reference values for cardiac output and organ blood volumes (23, 24).

These blood dose models have been used to demonstrate how the dose to the patient’s circulating blood depends not only on hemodynamic data but also on treatment modality, beam delivery parameters such as field size, treatment time, fractionation, and dose. While they have been able to show trends in RIL, their main weakness is that the results from blood dose simulations do not necessarily translate directly to doses to circulating lymphocytes, which may have different transition and flow parameters than the blood. Unfortunately, these are more complex and not as well-known (91, 92).

Jin et al. (93) developed a lymphocyte trafficking model that is an extension of an algorithm discussed previously (21). The framework considers 5 compartments of the immune system, i.e. the circulating blood, the bone marrow, specific lymphatic organs such as spleen, lymph nodes/vessels, and other lymphatic tissues in non-lymphatic organs such as gut, lung, liver and skin. Circulating and noncirculating lymphocytes are considered separately. The model also incorporates lymphocyte radiosensitivity and reproductivity. The authors assume that lymphocytes in the blood circulate at a higher rate than the blood. Clinical beam delivery times were not taken into account as the irradiation time was assumed to be equal to the blood circulation time, and all organs were treated as homogeneous.

To study the interaction between immunotherapy and radiotherapy, Friedrich et al. introduced a biophysical model of lymphocyte trafficking that takes into account primary and distal tumor masses, immune cell kinetics targeting tumor cells, and immune cell replenishment after radiation (94). Model parameters were derived from mouse data. The model suggests that the immune response is stronger when checkpoint inhibitors are administered at the time of radiation or shortly thereafter. It predicts that there is a window for radiotherapy that optimally balances radiogenic immune response and depletion of the immune cell pool.

In order to understand the impact of high-dose rate irradiation on the dose to the circulating blood and lymphocytes an algorithmic model was developed by Cucinotta and Smirnova (95). The model also incorporates a one-target-one-hit model of radiation-induced damage as a basis to consider the response of blood lymphocytes to the radiation exposure. It considers time-dependent dose delivery, radiosensitivity and concentration of lymphocytes, as well as blood flow characteristics through the blood circulatory system including the total blood volume and heart rate. The model confirms that the level of surviving blood lymphocytes increases as the dose rate increases.

## 3 Radiosensitivity of lymphocytes

Monocytes and macrophages isolated from peripheral blood cells are highly radioresistant (96, 97). Monocytes do not express proteins required for non-homologous end-joining and are impaired in base excision repair, which is likely to limit repair especially at higher doses (98). When monocytes proliferate into macrophages and dendritic cells, proteins are upregulated that make these cells repair competent. Dendritic cells are thought to be highly resistant to radiation-induced apoptosis (99). However, the irradiation of dendritic cells may impair their ability to stimulate T cells (100).

Peripheral blood lymphocytes are primed to undergo apoptosis (101). While most mammalian cells are radioresistant at rest and radiosensitive during proliferation, the opposite is true for circulating lymphocytes. Even a small amount of DNA damage appears to be sufficient to activate a DNA damage response and apoptosis (102). Damage to peripheral lymphocytes (e.g., chromosome aberrations) has been used as bio-dosimeters to predict late radiation toxicity in radiation therapy patients (103).

The literature discussed in the following sections is not always consistent in terms of notation. Naïve T cells can be categorized into helper T_h_ cells (CD3+, CD4+) and cytotoxic T_cyt_ cells (CD3+, CD8+) with regulatory T_reg_ cells (CD4+ CD25+, Foxp3+) as a subset of T_h_ cells. Categorization can also be done into naïve, effector T_eff_ (CD25+), and memory T cells (effector memory T_EM_ (CD45RO+, CD25-, CCR7-) and central memory T_CM_ (CD45RO+, CD25+, CCR7+)). Naïve B cells (CD27-) and B cells (CD19+, CD20+) can also play an immune-suppressive role, for example by blocking the T_cyt_ cell response. Naïve NK cells (CD16-) can become effector, regulatory, and memory NK cells (CD16+, CD56+, CD3-). NKT cells are a subset of T cells that express both CD3+ and CD56+.

### 3.1 Lymphocyte radiosensitivity studies in humans

The results of *in vivo* radiosensitivity studies in patients with qualitative or quantitative information are summarized in **Tables 1A, B** with the latter showing estimated alpha values [Gy^-1^] for a linear dose-response curve (exp(-αD)). A rather comprehensive study of lymphocyte radiosensitivity was performed by Trowell et al. already in 1952 (110). After whole-body irradiation, lymphocytes were counted in lymph nodes. In addition, lymph nodes and blood samples were irradiated *in vitro*. In 1975, Heier et al. (1) analyzed early and late T cell and B cell counts in patients with seminoma testis. B cells seemed to be more radiosensitive. They also concluded that B cells recovered more rapidly than T cells after the irradiation of the iliac and paraaortic lymph nodes and that irradiation of the thymus did not alter lymphocyte recovery.

**Table 1A:** Ranking of lymphocyte radiosensitivity based on lymphocyte depletion in patients.

Radiosensitivity Ranking	Dose Range	Time	Reference
B > T	40 Gy (20 fractions)	12 d – 10 y	(1)
CD4+ > CD8+	Therapeutic		(104)
B > T > NK, CD34+	0-2 Gy	6 h	(105)
B > T_h_; T_cyt_ > NK	50 Gy (25 fractions)	5 w	(106)
B > T; T_naive_ > T_memory_	26 Gy (13 fractions)	11 d – 4 m	(107)
CD4+ > CD8+ > pre-curser NK > NK, T_reg_	50-60 Gy (3-5 fractions)		(46)
CD4+ > CD8+	50-60 Gy (5-10 fractions)		(108)
B > T, NK	12 Gy (6 fractions)		(97)

**Table 1B:** Estimated alpha values (in Gy^-1^) for a linear dose-response curve exp(-aD) based on lymphocyte depletion in patients (fits performed using LMfit in python).

T_h_	T_cyt_	B	NK	Peripheral lymphocytes	Reference
0.45 +/- 0.02	0.46 +/- 0.03	0.67 +/- 0.03	0.37 +/- 0.03		(106)
				0.40 (0.08 – 2.0)	(93)
				0.58 (0.28 – 1.23)	(118)

Lymphocyte radiosensitivity *in vivo* was evaluated by Clave et al. (105) based on whole-body irradiation of patients prior to bone marrow transplantation. Lymphocyte subpopulations were counted after irradiation at 2 Gy/fraction. B cells were the most sensitive, followed by T cells (CD4+, CD8+) and NK cells. CD34+ progenitor cells appeared to be highly radioresistant. Note that the easurements include circulating lymphocytes while also irradiating lymphatic vessels. A similar study by Girinsky et al. found no statistically significant difference in radiosensitivity between T cells and B cells (111). Lymphocyte depletion and recovery for different subpopulations has also been studied for low dose whole body irradiation from the Chernobyl accident and in atomic bomb survivors (112).

B cells were the most sensitive and NK cells the least sensitive lymphocyte fraction in cancer patients receiving pelvic radiation therapy (106). No significant differences between T_h_ cells and T_cyt_ cells were reported. The counts of the lymphocyte subpopulation as a function of total body dose can be translated into alpha values in a linear dose-response curve. Belka et al. (107) evaluated lymphocyte subpopulations after radiation therapy and found that B cells and T cells seemed to be most affected. Recovery of CD8+ cells was significantly faster than that of CD4+ cells, and naïve cells were generally more sensitive than memory cells. Lymphocytes were still unable to respond adequately to antigen stimulation even after recovery of the population.

A comprehensive assessment of circulating immune cell populations in response to stereotactic body radiation therapy in patients with liver cancer was performed by Gustafson et al. (46). They found a severe decrease (~50%) in T cells in liver SBRT patients, even in the absence of bone marrow or nodes in the field, with CD4+ cells being most affected, while CD8+ cells showed no significant differences compared to pre-treatment levels. More specifically, within the CD4+ compartment, T_reg_ cells were not affected. SBRT did not appear to affect mature NK cells (CD16+) but did affect pre-cursor cells (CD16-). McGee et al. analyzed the blood of 31 patients after stereotactic ablation radiation therapy (113). They showed that the effect of radiation on T cells and NK cells depends on the treatment site. Therapy of parenchymal sites induced a systemic immune response (i.e., a decrease in NK cells and an increase in memory CD4+ and CD8+ T cells). This was not seen in non-parenchymal sites (bone and brain).

Zhao et al. (108) analyzed lymphocyte subpopulations after SBRT of early-stage lung cancer. The number and relative percentage of CD4+ T cells were significantly decreased, whereas the number of CD8+ T cells was less affected as their relative percentage was almost unchanged. This decreasing ratio of CD4+/CD8+ T cells was also observed by Yang et al. (104) in head and neck cancer patients. The change in the ratio could not be explained by the small difference in radiosensitivities of CD4+ T cells and CD8+ T cells but was presumably caused by radiation-induced priming and mobilization of CD8+ T cells compensating for the loss of CD8+ T cells. Lymphocyte subpopulations in patients after radiation therapy have also been studied in other sites, such as in prostate cancer (42, 114–116) and in breast cancer (117, 118), demonstrating radiosensitivity of B cells in particular.

Heylmann et al. (97) analyzed T cells and monocytes after treatment in leukemia patients receiving whole body irradiation (6 times 2 Gy). Monocytes showed high radioresistance, and the difference in the lower response between T cells and NK cells was not statistically significant. Three patients who received 12 Gy in 3 days (2 times 2 Gy per day) were analyzed. Analysis of γH2AX foci indicated efficient elimination of damaged B cells during treatment. In NK cells (CD56+), DNA damage accumulated in the surviving NK after repeated irradiation. Whether these cells later undergo apoptotic death or survive in the presence of DNA damage was unclear.

Assuming an exponential dose response relationship, the alpha value of circulating lymphocytes has also been deduced indirectly in patients. A lymphocyte trafficking model was fitted to 51 patients with abdominal cancer treated with radiotherapy (93). The patient specific α values had a median of 0.40 Gy^-1^ (range 0.08 – 2.0 Gy^-1^). Similarly, for hepatocellular carcinoma the dose to circulating lymphocytes was estimated using a dynamic blood circulation model (23) and combined with the observed lymphocyte depletion in patients, empirically accounting for both cell death and lymphocyte replenishment. The *in vivo* derived patient-specific α had a median value of 0.58 Gy^-1^ (range 0.28 - 1.23 Gy^-1^) (109).

Schaue et al. (119) isolated lymphocytes from colorectal and prostate cancer patients before, during, and one week after chemoradiation therapy. In most patients, they found an increase of T_reg_ cells as well as CD8+ cells after radiation which was more pronounced in colorectal patients. A relative resistance of T_reg_ could have negative consequences in radiation therapy of their tumor protective role as compared to the immune stimulatory role of more radiosensitive T_cyt_ cells and T_h_ cells. However, radiation can also reduce protein expression and reduce functionality of T_reg_ cells (120).

### 3.2 Lymphocyte radiosensitivity studies in mice

**Figure 1:**
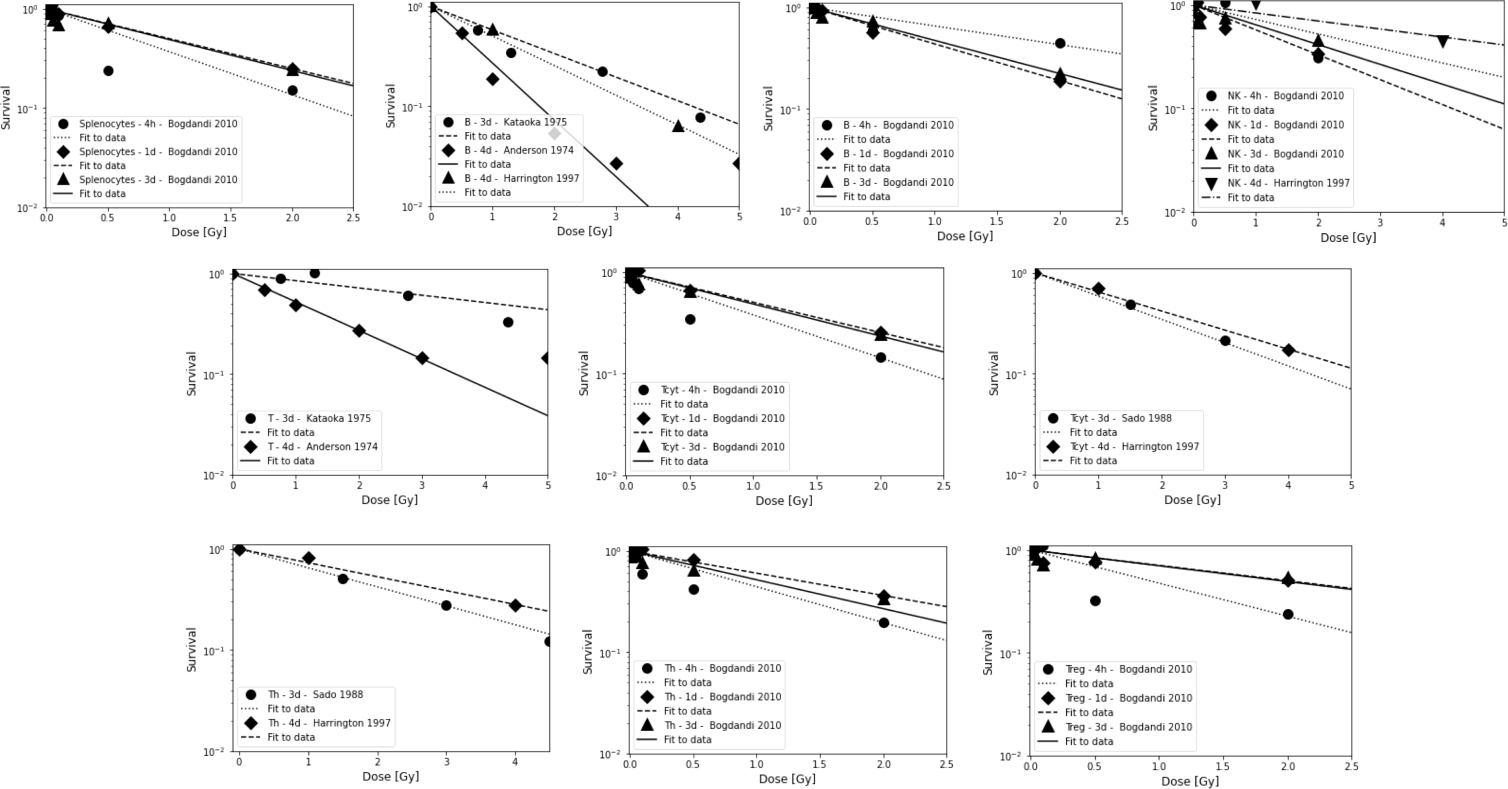
Radiosensitivity of lymphocyte sub-populations in mice for studies shown in **Table 2B**. First row: Splenocytes, B cells, and NK cells. Second row: Combined T cells and T_cyt_ cells. Third row: T_h_ cells and T_reg_ cells. Data points are shown up to 5 Gy but alpha value fits were only done for data points ≤3 Gy because lymphocytes will not receive more than the prescription dose in a single fraction in radiation therapy, and because the majority of the dose-response data show a more shallow slope and a saturation at higher doses. The data points were extracted from the published figures (using plotdigitizer (plotdigitizer.com)). Experimental error bars are not shown but are included in the fits (performed using LMfit in python).

Results from preclinical studies of *in vivo* radiosensitivity with qualitative or quantitative information are summarized in **Tables 2A, B** and include fitting of exponential dose-response curves where possible (**Figure 1**).

**Table 2A:** Ranking of lymphocyte radiosensitivity based on lymphocyte depletion in mice.

Radiosensitivity Ranking	Dose Range	Time	Reference
B > T; resting T > activated T	0 - 10 Gy	4 d	(121)
B > T	0 - 100 Gy	3 d	(122)
B > T	0.5 - 15 Gy	6 d	(123)
CD8+ > CD4+	0 - 10 Gy	3 d	(124)
B > CD8+ > CD4+ > NK	0 - 7 Gy	1, 4, 7 d	(125)
B > CD8+ > CD4+ > NK	1 - 7 Gy	1, 4, 7 d	(126)
B > T > NK; CD4+ > CD8+	3 Gy		(127)
T_naive_ > T_memory_ (CD8+)	2 - 6 Gy	4 - 60 d	(128)
B > T (CD4+)	8 Gy	4 h - 12 d	(129)
Spleen; B > CD8+ > CD4+ > T_reg_ > NK	0.01 - 2 Gy	4 h - 7 d	(130)
Total, spleen, lymph nodes: CD8+ > CD4+ > T_reg_	2, 5 Gy	0.5, 5, 15 d	(131, 132)
T_reg_ > T_mem_	1.25 Gy		(52)
CD8+ T; CD44^lo^ T_naive_ > CD44^hi^ T (NKT and T_mem_)	10 Gy	24, 48, 72, 120 h	(133)
T_reg_ radioresistant	0 - 20 Gy		(134)
Spleen; CD4+ > T_reg_	2 Gy	4 h – 11 d	(135)
T_naive_ > CD8 T_EM_ > CD8 T_cm_	0 - 4 Gy	72 h	(136)
Cardiac: B > CD8+ > CD4+; Spleen: B > CD8+ = CD4+	2 Gy/day		(137)
CD8+ circulating > CD8+ infiltrating CD8+ nodes, spleen > CD8+ gut	0 - 20 Gy	24 h	(138)
Circulating > Splenic	6, 12 Gy	1, 4, 7 d	(139)

**Table 2B:** Estimated alpha values (in Gy^-1^) for a linear dose-response curve exp(-aD) based on lymphocyte depletion in mice (fits were performed using LMfit in python and include only data points ≤ 3 Gy).

T_h_	T_cyt_	T	T_reg_	B	NK	Spleen	T	Ref.
		0.65+/-0.01		1.31+/-0.07			4 d	(121)
		0.17+/-0.03		0.54+/-0.02			3 d	(122)
0.43+/-0.01	0.53+/-0.01						3 d	(124)
0.32+/-0.01	0.44+/-0.01			0.68+/-0.02	0.18+/-0.04		4 d	(125)
0.82+/-0.06	0.97+/-0.03		0.74+/-0.10	0.42+/-0.22	0.32+/-0.25	1.00+/-0.15	4 h	(130)
0.51+/-0.04	0.69+/-0.02		0.34+/-0.05	0.83+/-0.01	0.55+/-0.04	0.69+/-0.01	1 d	(130)
0.66+/-0.09	0.73+/-0.05		0.35+/-0.11	0.75+/-0.01	0.44+/-0.09	0.72+/-0.06	3 d	(130)

Anderson et al. (121) investigated the effect of radiation on lymphocyte migration. Activated thoracic duct lymphocytes from CBA inbred mice were used as a surrogate for T cells (80-85% T cells, 15-20% B cells) and those from athymic (nude, nu/nu) mice were used as a surrogate for B cells (97%). B cells were highly radiosensitive compared to T cells and activated T cells were more radioresistant than their resting counterparts. In another study, T cells were shown to be more radioresistant than B cells in the spleen of C3Hf mice (122). After whole-body irradiation, T and B cells were analyzed after 3 days. At low doses (0.47 Gy), the number of T and B cells in the spleen was significantly higher compared to unirradiated control mice.

That B cells are more radiosensitive than T cells was also observed after whole-body irradiation with doses of 0.5-15 Gy (123). Mice were sacrificed and thymus, spleen, mesenteric lymph nodes, femur, tibia and fibula were removed, and peripheral blood was analyzed after 6 days. The authors also studied recovery of T and B cell populations after 6 Gy and showed that cells in the thymus and spleen recovered more rapidly than those in the lymph nodes and in the bone marrow. Hochman et al. (140) reported the relative resistance of NK cells in the spleen of (C57BL/6 x C3H/He)F mice and the temporary cessation of progenitor activity. Sado et al. (124) showed that cells from C3H mice were more radioresistant compared to BALB/c, C57BL/6, and B10.BR mice. After whole-body irradiation, T cells were analyzed in the spleen after 3 days. CD8+ T cells were slightly more radiosensitive than CD4+ T cells.

Harrington et al. (125) irradiated C57Bl/6 mice with doses of 0-7 Gy (whole-body) and analyzed splenic T cells (CD4+ and CD8+), B cells, and NK cells after 1, 4, and 7 days after irradiation. They observed a 7-fold enrichment of NK cells and a 3-fold enrichment of T CD4+ cells, while the proportion of CD8+ cells was unchanged and B cells decreased. While radiation may reduce the total number of lymphocytes, the spleen may be enriched when comparing subpopulations. B cells were most sensitive to radiation, followed by CD8+, CD4+, and NK cells. In a study by Chambers et al. (126) on lymphocyte subpopulations after whole-body irradiation of mice, the total number of peripheral lymphocytes decreased as a function of dose and the lymphocyte distribution changed. Relative to the total number of lymphocytes, CD8+ increased slightly on day 1 and then decreased, while CD4+ increased 2-fold on day 4 after 7 Gy. The relative contribution of NK cells increased 9-fold at day 4 at 7 Gy, while the relative number of B cells decreased at all dose levels, e.g., by half at 1 Gy. This indicated radioresistance of NK cells relative to CD4+, CD8+, and B cells.

Mice were exposed to a whole-body dose of 3 Gy of protons and ^60^Co X-rays by Kajioka et al. (127) and acute effects on the immune system were assessed. Overall, B cells were the most sensitive cell population, while T cells were moderately sensitive and NK cells were the most resistant cell population. Within the T cell population, T_h_ cells were more resistant than T_cyt_ cells. This was also true for the splenic lymphocyte population. B cells had the most rapid recovery and recovered completely in the spleen but not in circulating lymphocytes. Grayson et al. (128) found that naive T cells were more sensitive than their memory counterparts (CD8+) after whole body irradiation of mice at 2-6 Gy. Lymphocytes were isolated from the spleen, lymph nodes, bone marrow, and peripheral blood. In a dose-dependent manner, memory CD8+ T cells were enriched in the spleen, increasing from 20% of the total CD8+ population in untreated mice to 76% after 6 Gy. Garg et al. (129) analyzed immune cell populations in the intestinal mucosa after whole body irradiation of mice and found that B cells were more sensitive compared to T cells.

In another study of apoptosis in mouse spleen cells, animals were sacrificed 4 h, 1, 3 or 7 days after irradiation (130). The authors analyzed T_h_, T_cyt_, T_reg_, NK, B, and CD8+CD44+ memory T cells. Low dose radiation decreased apoptosis compared to the control. In terms of apoptosis at 4h, CD8+ and B cells were more resistant to low doses but were very sensitive to 2 Gy, while NK cells and T_reg_ were much more resistant to higher doses. B cells were the most sensitive, followed by T_cyt_, T_h_, T_reg_, and NK cells. Analysis of subpopulations after 7 days showed that T_cyt_ cells started to regenerate earlier than T_h_ cells.

In a series of investigations, Qu et al. compared the radiosensitivity of CD4+CD25^high^ Foxp3+ T_reg_ cells and CD4+CD25- T cells in mice after 2 Gy (131) and 5 Gy (132) whole-body irradiation. *In vivo* depletion showed an increased sensitivity of CD8+ compared to CD4+, while the level of CD4+CD25^high^ T_reg_ increased. For both spleen and lymph nodes, the radiosensitivity of CD8+ was higher than CD4+, followed by T_reg_ cells. In the thymus, the levels of CD4+CD8+ decreased. However, the newly developed T_reg_ cells in the thymus were less sensitive to radiation than other thymocytes. The function of Treg cells was impaired after 5 Gy radiation but not after 2 Gy, suggesting a threshold effect.

Assessing lymphocyte populations after low-dose total body irradiation in mice, Liu et al. found significant decrease in the T_reg_ cell population (52), but an increase in memory T cells (CD4+/CD8+). Despite increased radiosensitivity, T_cyt_ cells were activated in mice after fractionated low-dose exposure (0.2 Gy), which was not previously observed for T_h_ cells (141). Spleen cells were analyzed after whole body irradiation of mice with 10 Gy in another report (133). Analysis was performed at 24, 48, 72, and 120 h. CD4+ T cells were significantly more resistant than CD8+ T cells, and CD44^high^ T cells, including NKT cells and memory T cells, were significantly more resistant than CD44^low^ (naive) T cells. Furthermore, the effect of radiation on naturally occurring T_reg_ cells was investigated in a mouse model (134). The number of T_reg_ cells increased after irradiation as they appeared to be more radioresistant compared to other lymphocytes. Their functional integrity was also unaffected. However, this observation could also be caused by radiation-induced T_reg_ cell activation.

Balogh et al. (135) irradiated C57Bl/6 mice with 2 Gy (whole body) and analyzed changes in lymphocyte fractions isolated from the spleen. T_reg_ cells were less prone to apoptosis than other lymphocytes after *in vivo* irradiation. The results showed a greater decrease in CD4+ numbers compared to T_reg_ cells that were not only less susceptible to radiation-induced apoptosis but also recovered faster than CD4+Foxp3- cells. However, irradiated T_reg_ cells were functionally compromised with a reduced suppressive capacity (~2.5 fold). In addition, radiation increased the proliferation rate of surviving CD4+ cells. In a study by Pugh et al. (136), mice were irradiated *in vivo* at doses up to 4 Gy and splenocytes as well as peripheral lymphocytes were analyzed at 3, 12, 17, and 24 h. CD8 T_EM_ cells were more resistant and naive T cells more sensitive. CD8 T_CM_ cells were significantly more resistant *in vivo* than *in vitro*. The authors hypothesize that this may be due to the genome-wide chromatin structure that governs early DSB binding and survival. Chromatin remodeling occurs during the differentiation of naive T cells to memory T cells.

In T cell recovery after low-dose whole body irradiation of female C57BL/6 mice, CD4+ T cell reconstitution was delayed more than that of CD8+ T cells (142). Venkatesulu et al. showed lymphopenia after heart (2 Gy per day for 5 days) and spleen (1 Gy per day for 5 days) irradiation of female BALB/c mice *in vivo* (137). B cells were most sensitive in both cohorts. For cardiac irradiation this was followed by CD8+, while CD4+ depletion was moderate in comparison. For splenic irradiation there was no significant difference between CD8+ and CD4+. Radio-resistance of splenic lymphocytes compared to circulating lymphocytes has also been shown in a C57BL/6J mouse model after studying partial body irradiation with and without lymph node involvement (139). The authors also investigated the effect of different field sizes using a small animal image-guided irradiation device.

A study by Arina et al. (138) in which mice were irradiated with a whole-body dose of 8 Gy showed a dose-dependent loss of circulating CD8+ T lymphocytes, but not of tumor-infiltrating CD8+ T cells after 24 h. The authors also quantified the sensitivity of parenchymal CD8+ in various organs. Within certain solid organs there was a higher radio-resistance compared to T cells in circulation and in lymphoid organs. Lymph nodes and spleen had the most radiosensitive CD8+ T cells, while CD8+ T cells in the intestine were the most radioresistant. They hypothesized that the higher radioresistance of parenchymal CD8+ T cells from non-lymphoid compared to lymphoid solid organs is due to the presence of tissue resident memory cells. In tissues harboring the most radioresistant CD8+ T cells (intraepithelial and tumor), not only cells with the standard memory T cells but all CD8+ T cells were similarly radioresistant. In contrast, memory T cells in the liver were more radiosensitive than other T cells.

### 3.3 *In vitro* lymphocyte radiosensitivity studies

**Figure 2:**
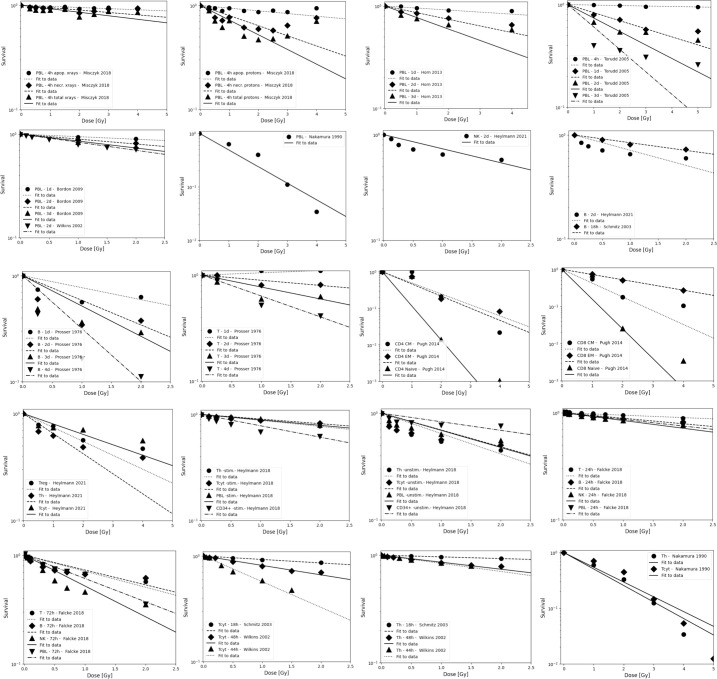
Radiosensitivity of peripheral lymphocytes, NK cells, T cells, and B cells. Data are grouped to illustrate both, differences between experiments as well as differences between subpopulations. Data points are shown up to 5 Gy but alpha values fits include only data points ≤ 3 Gy because lymphocytes will not receive more than the prescription dose in a single fraction in radiation therapy, and because the majority of the dose-response data show a more shallow slope and a saturation at higher doses. The data points were extracted from the published figures (using plotdigitizer (plotdigitizer.com)). Experimental error bars are not shown but are included in the fits (performed using LMfit in python).

Results from *in vitro* radiosensitivity studies with qualitative or quantitative information are summarized in **Tables 3A, B** and include fitting exponential dose response curves where possible (**Figure 2**).

**Table 3A:** Ranking of lymphocyte radiosensitivity based on *in vitro* studies.

Radiosensitivity Ranking	Dose Range	Time	Reference
B > T	0 - 10 Gy	24, 48, 72, 96 h	(143)
B > T	0 - 4 Gy	96 h	(144)
NK = T	0 - 30 Gy	4 h	(145)
NK (CD56+, CD16+) > NK (CD56+)	0 - 30 Gy	3, 48 h	(146)
CD4+ = CD8+	0 - 5 Gy		(147)
T; patient variation			(148)
NK > CD8+, B > CD4+	15 Gy	48 h	(149)
B > CD4+ > CD8+ > NK	2 Gy	24 h	(150)
CD8+ > CD4+	2, 9 Gy	48 h	(151)
CD8+ > CD4+	0 - 2 Gy	48 h	(152)
NK > CD8+ > B > CD4+	0 - 1.5 Gy	44 h, 68 h	(153)
B > CD8+ > CD4+; T_h_ (male) > T_h_ (female)	0 - 2 Gy	18 h	(154)
CD34+CD38- stem > CD34+CD38+ differentiated	5 Gy	4 h, 16 h	(155)
Peripheral lymphocytes	0 – 15 Gy	4, 24, 48, 72 h	(156)
Peripheral lymphocytes	0 - 8 Gy	24, 48, 72 h	(157)
CD34+CD38- stem > CD34+CD38+ progenitors	3 Gy	0.5 – 6h	(158)
T_reg_ (CD4+CD25+) > T (CD4+CD25-)	0 - 2 Gy		(159)
B > CD8+ > CD4+	0 - 8 Gy	24, 48, 72 h	(160)
NK (CD56+, CD16+) = NK (CD56+)	0 - 80 Gy	2 – 72 h	(161)
T(non-prof) > T(prof); CD34+(non-prof) = CD34+(prof); T_h_ > T_cyt_ > CD34	0 – 2 Gy	6 – 48 h	(96)
NK > B > T	0 – 60 Gy	24, 48, 72 h	(162)
protons vs. photons	0 - 4 Gy	1 h, 4 h	(75)
CD4+ > T_reg_	0, 10 Gy	48 h	(120)
T(non-prof) > B > T > NK > CD34; T_h_ > T_reg_ > T_cyt_	0 – 8 Gy	1 – 24 h	(97)
CD4+CD25- T > CD4+CD25^high^ Foxp3+ T_reg_	5 Gy	12 h	(131, 132)
CD8+ > CD4+; T_CM_, T_naive_ > T_EM_	1 - 10 Gy	3 - 24 h	(136)

**Table 3B:** Estimated alpha values (in Gy^-1^) for a linear dose-response curve exp(-aD) based on in vitro measurements (fits were performed using LMfit in python and include only data points ≤ 3 Gy).

T_p_	T	T_cyt_	T_h_	T_reg_	Time		Reference
	0.00 +/- 0.02				24 h		(143)
	0.11 +/- 0.02				48 h		(143)
	0.26 +/- 0.05				72 h		(143)
	0.45 +/- 0.03				96 h		(143)
	0.77						(163)
	0.65						(163)
	0.05 +/- 0.01				24 h		(162)
	0.34 +/- 0.03				72 h		(162)
		0.61 +/- 0.05	0.68 +/- 0.02				(147)
		0.21 +/- 0.01	0.15 +/- 0.02		48 h		(152)
		0.56 +/- 0.04	0.17 +/- 0.01		44 h		(153)
		0.08 +/- 0.01	0.04 +/- 0.01		18 h		(154)
		0.22 +/- 0.05	0.43 +/- 0.08	0.30 +/- 0.04	24 h		(97)
0.18 +/- 0.03		0.36 +/- 0.03	0.44 +/- 0.06		24 h		(96) unstim
0.25 +/- 0.02		0.10 +/- 0.01	0.13 +/- 0.01		24 h		(96) stim.

T_CM_ CD4+	T_EM_ CD4+	T_naive_ CD4+	T_CM_ CD8+	T_EM_ CD8+	T_naive_ CD8+	Time	Reference
0.69 +/- 0.17	0.76 +/- 0.14	2.14 +/- 0.07	0.85 +/- 0.03	0.32 +/- 0.01	1.84 +/- 0.07	72 h	(136)

B	NK		General Peripheral Lymphocytes		Time		Reference
0.26 +/- 0.06					24 h		(143)
0.53 +/- 0.10					48 h		(143)
0.66 +/- 0.10					72 h		(143)
1.15 +/- 0.13					96 h		(143)
0.17 +/- 0.01					18 h		(154)
0.34 +/- 0.07	0.31 +/- 0.04				24 h		(97)
0.12 +/- 0.01	0.17 +/- 0.01		0.14 +/- 0.01		24 h		(162)
0.31 +/- 0.05	0.65 +/- 0.07		0.49 +/- 0.02		72 h		(162)
	0.08 +/- 0.01				18 h		(161)
			0.72 +/- 0.04				(147)
			0.18 +/- 0.01		48 h		(152)
			0.01 +/- 0.01		4 h		(156)
			0.18 +/- 0.01		24 h		(156)
			0.30 +/- 0.02		48 h		(156)
			0.50 +/- 0.04		72 h		(156)
			0.06 +/- 0.01		24 h		(157)
			0.11 +/- 0.01		48 h		(157)
			0.15 +/- 0.01		72 h		(157)
			0.05 +/- 0.01		24 h		(160)
			0.15 +/- 0.02		48 h		(160)
			0.26 +/- 0.04		72 h		(160)
			0.37 +/- 0.04		24 h		(96) unstim
			0.12 +/- 0.01		24 h		(96) stim.
			0.08 +/- 0.02		4 h		(75) X-rays
			0.32 +/- 0.04		4 h		(75) protons
			0.45 [0.05-1.2]				(148)

The survival of unstimulated T and B cells from a healthy donor was evaluated in 1-day intervals up to 4 days after irradiation with doses up to 10 Gy by Prosser et al. (143). They observed a higher radiosensitivity of B cells compared to T cells. Cole et al. measured T cell survival in blood from 9 donors and T cell lines (163). The *in vitro* survival of human peripheral blood lymphocytes and thymocytes (T cell progenitors) was also measured after 4 days in a study by Kwan and Norman in healthy volunteers (144). B cells appeared to be slightly more radiosensitive than T cells. The authors concluded that there are subpopulations of T and B cells with different radiosensitivities, resulting in a biphasic survival curve for T cells. Brovall et al. (164) studied NK cell activity in the peripheral blood of healthy adults. While activity was lost at 30 Gy, it was enhanced at lower doses (5 to 20 Gy, depending on the donor). This suggests that radiation affects the cytotoxic function of NK cells before death or apoptosis is observed. Zarcone et al. (145) investigated the effect of radiation on different NK cell activities. The cytotoxic functions of NK and T cells showed identical sensitivity to radiation. Similarly, Rana et al. (146) investigated cytotoxic activities of NK cells as a function of dose up to 30 Gy and showed that CD16+ were the most radiosensitive.

Nakamura et al. (147) investigated the radiosensitivity of proliferating T_h_ and T_cyt_ lymphocytes *in vitro* using a colony formation assay. This particular study is often cited when discussing lymphopenia and its relationship to lymphocyte radiosensitivity. Strikingly, the measured cell survival curves follow a linear-quadratic dose-response fitted by the linear quadratic model (PBL: α=0.29+/-0.01; β=0.14+/-0.01; CD4+ α=0.32+/-0.01; β=0.13+/-0.01; CD8+ α=0.19+/-0.03; β=0.14+/-0.01), whereas in the majority of *in vitro* studies reviewed, the response curves show either an exponential or upward-sloping curve and a decrease in response (saturation) at higher doses.

Using blood from cancer patients and healthy individuals, Geara et al. analyzed the radiosensitivity of peripheral T lymphocytes *in vitro* and demonstrated a significant variation among individuals (148). Patient-specific α values were fitted with a median of 0.45 Gy^-1^ (range 0.05 - 1.20 Gy^-1^). Seki et al. (149) showed that CD8+ T cells were more susceptible to interphase death than CD4+ T cells and NK cells were the most radiosensitive. Philippe et al. (150) assessed apoptosis after 24 h *in vitro*. B cells showed more apoptotic cells than T cells. Among T cells, T_h_ cells were the most sensitive, followed by T_cyt_ cells. NK cells were the most resistant. Spontaneous apoptosis in immune subsets of *in vitro* cultured cells correlated with differences in radiation induced apoptosis. Radojcic and Crompton used peripheral lymphocytes from three donors to assess the age dependence of CD4+ and CD8+ cell apoptosis at 2 and 9 Gy and suggested that radiosensitivity may be higher in younger individuals (151). CD8+ were more sensitive than CD4+.

Wilkins et al. studied the apoptotic response in lymphocytes using blood from healthy volunteers. One study focused only on CD8+ and CD4+ cells (152). CD8+ T cells were more sensitive to radiation-induced apoptosis than CD4+ at doses up to 2 Gy at 48 h. The authors state that the relative amounts of CD4+ and CD8+ in the combined culture likely influenced the observed apoptosis due to changes in the production of specific cytokines in the cell culture. A second study examined B cells, NK cells, and CD4+ and CD8+ T-cells at 44 h and 68 h after exposure to up to 1.5 Gy (153). Although B cells showed the highest radiation-induced apoptotic response at 1 Gy, CD8+ T-cells appeared to be the most sensitive based on their low spontaneous apoptotic fraction. At 48 h, the radiation-induced apoptosis of the cell subpopulations decreased in the order of NK cells, CD8+ T cells, B cells and CD4+ T cells, although the differences were not significant. Again, lymphocytes in isolation appeared to be more responsive to radiation than those cultured in the presence of other lymphocytes.

In a study of spontaneous and radiation-induced apoptosis of human lymphocytes *in vitro*, lymphocytes from females were less radiosensitive compared to those from males and radiosensitivity seemed to increase with age (154). T_cyt_ cells were more sensitive than T_h_ cells. Hayashi et al. (155) investigated radiation-induced apoptosis of stem/progenitor cells in human umbilical cord blood. The CD34+/CD38− stem cell population was more sensitive to radiation-induced apoptosis, compared to more differentiated CD34+/CD38+ and CD34−/CD38+ cells. Human lymphocytes were irradiated *in vitro* with doses up to 15 Gy by Torudd et al. (156). Apoptosis was assessed at 4, 24, 48, and 72 hours. There was very little effect at the early time point at 4 hours. In the context of establishing a predictor of patient’s response based on individual lymphocyte radiosensitivity, Bordon et al. (157) evaluated how radiation induced apoptosis correlates with late toxicity and patient’s radiosensitivity in cervical cancer. Radiation-induced apoptosis was analyzed at 24, 48, and 72 hours.

Milyavsky et al. (158) reported that human hematopoietic stem cells (CD34+) exhibited delayed DNA double-strand break rejoining, persistent γH2AX foci, and increased apoptosis after irradiation compared to progenitor cells. Cao et al. (159) compared the radiosensitivity of T_reg_ cells (CD4+CD25+) and effector T cells (CD4+CD25-) *in vitro* using lymphocytes from healthy individuals and hepatocellular carcinoma patients. In the range of 0-2 Gy, Treg cells were more radiosensitive than effector T cells, the opposite trend compared to a previous *in vivo* study (135). T_reg_ cell functionality was moderately affected in Cao et al. (165) using *in vitro* cultured and *in vitro* irradiated T_reg_ cells, showing a dose-dependent reduction in T_reg_ cell proliferation as well as an alteration in phenotype.

In another study of peripheral lymphocytes from healthy donors, radiation-induced apoptosis *in vitro* was not apparent until 24 h after exposure when data were analyzed at 24, 48, and 72 h (160). Radiosensitivity was highest for B cells, followed by T_cyt_ cells, and T_h_ cells, but the trend was reversed for B cells and T_cyt_ cells after 48h and 4 Gy. Hietanen et al. (161) applied single and fractionated doses to enriched NK cell populations. Cell survival was reported from 2 to 72 h and for doses up to 80 Gy. The response based on the reported α values was very similar for CD16+ and general CD56+ cells at 18 h at doses up to 40 Gy.

A review of the radiosensitivity of human and murine peripheral blood lymphocytes concluded that stem cells, T_h_ cells, T_cyt_ cells, monocytes, neutrophils and, to a high degree, B cells exhibit a radiosensitive phenotype, whereas T_reg_ cells, macrophages, dendritic cells and NK cells appear to be more radioresistant (166). The same authors studied stimulated (proliferating) and unstimulated (non-proliferating) peripheral lymphocytes in the blood from healthy volunteers (96). Unstimulated peripheral lymphocytes contained mainly T cells arrested in G0/G1. Upon stimulation of the CD3 T-cell receptor and the CD28 co-receptor with anti-CD3 and anti-CD28, respectively, the cells begin to proliferate. Lymphocytes were shown to be highly radiosensitive but stimulation induced radioresistance in several T cell subsets, with the exception of CD34+ cells which did not become radioresistant when stimulated to proliferate. There was no difference in repair between stimulated and unstimulated cells, i.e., the difference in radiosensitivity was likely caused by the induced DNA damage. The investigators found that most of the cells underwent apoptosis with only a small fraction of necrosis, with data collected between 6 and 48 hours after irradiation. It was concluded that T cells and B cells are highly sensitive and undergo apoptosis at doses as low as 0.125 Gy with no apparent threshold and a saturation of ~50% at about 1-2 Gy. Sensitivity was highest for non-proliferating T cells followed by B cells, and NK cells. However, while non-proliferating T and B cells were sensitive, they had a high repair capacity, which was also the case for CD34+. There was no significant difference in radiosensitivity between the non-proliferating T cell subcategories. The same authors then measured the *in vitro* dose response of blood cells from healthy volunteers (97). The analysis included unstimulated T cells (T_reg_, T_h_, T_cyt_) purified with magnetic beads as well as unstimulated B cells, and NK cells obtained from peripheral blood. Doses ranging from 0.5 to 8 Gy were administered. T_h_ cells were the most sensitive (30% apoptosis level at 0.5 Gy, while T_reg_, NK and B cells showed values around 20–25%). The authors point out that absolute numbers may be associated with uncertainties because there may be early apoptotic events that have been fragmented, or late apoptotic events that have not yet materialized at the time of the assay.

Apoptotic cells may lose membrane integrity and become secondary necrotic cells that retain immune activation properties. Falcke et al. (162) studied lymphocyte cell death by apoptosis, primary necrosis, and secondary necrosis (late apoptotic). They found that B cells and NK cells died mainly by apoptosis (secondary necrosis), whereas T cells showed significant primary and secondary necrotic cells. NK cells were the most sensitive to radiation, followed by B cells and T cells. The researchers also analyzed cell viability. In an *in vitro* study of human peripheral lymphocytes, necrosis was more frequent than apoptosis, especially with proton irradiation (75). This may indicate a mechanistic difference in lymphocyte damage when comparing photon and proton radiation (75, 167). The accumulation of radiation-induced repair protein foci differed after proton versus X-ray irradiation (168). Annexin V labeling was performed 1 h and 4 h after irradiation with doses of 0-4 Gy (75). Alpha values for peripheral lymphocytes differed between X-rays and protons as well as between apoptosis and necrosis (apoptosis X-rays: α=0.02+/-0.01 Gy^-1^; apoptosis protons: α=0.03+/-0.01 Gy^-1^; necrosis X-rays: α=0.04+/-0.01 Gy^-1^; necrosis protons: α=0.15+/-0.03 Gy^-1^).

Using blood from healthy volunteers Beauford et al. (120) found that T_h_ cells were more radiosensitive than T_reg_ cells. Although T_reg_ cells appeared to be more resistant, radiation caused a decreased Foxp3 expression as well as decreased expression of CD25 and CTLA-4, resulting in a reduced ability to suppress CD8+ T cell proliferation. Vandevoorde et al. (169) compared the dose response of CD34+ cells and umbilical cord T cells from newborns and adults. Naïve and memory T cells were analyzed *in vitro* 0.5 h after irradiation with low doses (100-200 mGy). Newborn peripheral T lymphocytes were significantly more radiosensitive than adult peripheral T lymphocytes. This may be due to immunophenotypic changes of T lymphocytes with age.

De Kruyff et al. (170) analyzed the functional behavior of lymph node T cells in mouse cell cultures as a function of dose. Specifically, the authors evaluated the helper activity of CD4+ T cells in terms of their ability to induce immunoglobin synthesis (IgG, IgM, and IgE synthesis) in B cells. The capacity for IgG synthesis was not affected, while that for IgE (which depends on IL-4 and IL-5) was significantly reduced. Thus, IL-4 in T_h_ cells appears to be sensitive to radiation, causing T cell functions to show large variations in radiosensitivity. Pugh et al. (136) measured the radiosensitivity of naïve lymphocytes, effective memory cells (CD8, T_EM_), and central memory cells (T_CM_) from mice *in vitro*. There was no significant difference in radiosensitivity between T cell subsets. However, CD8 T_EM_ cells were more radioresistant and showed less interphase death than T_CM_ cells or naïve T cells. CD4 T cells were more radioresistant than CD8 T cells. This pattern was extended to both CD4 naïve T cells and T_CM_ cell subsets. It was unclear whether the enhanced radioresistance of T_reg_ cells could fully account for the enhanced radioresistance of any specific CD4 subset.

Qu et al. compared the radiosensitivity of CD4+CD25^high^ Foxp3+ T_reg_ cells and CD4+CD25- T cells *in vitro* showing higher sensitivity for CD4+CD25- T cells than for CD4+CD25^high^ T_reg_ cells at 2 Gy (131) and 5 Gy (132), respectively. They reported that more dead cells were observed in the T_eff_ cell population than in the T_reg_ cell pool, which correlated with a higher levels of anti-apoptotic protein expression in T_reg_ cells. They also found that the T_eff_ cell suppressive capacity of the *in vitro* irradiated T_reg_ cells was only moderately affected by radiation. The evaluation was performed 2 weeks after irradiation.

## 4 Summary and discussion

The radiosensitivity of lymphocytes has been evaluated in a variety of ways, including studies in humans, pre-clinical studies, and *in vitro* clonogenic cell survival assays. The available data published in the open literature have been reviewed in this work. Interpretation of experimental data is often difficult. For example, *in vitro* measurements must be corrected for spontaneous apoptosis, which is particularly relevant for B cells. In addition, data from *in vivo* studies have to take into account that a part of the lympho-hematopoietic system has been irradiated, allowing for lymphocyte redistribution from non-irradiated areas. Expression changes may also occur.

Studies based on lymphocyte depletion in patients consistently suggest that B cells are the most radiosensitive, followed by T cells and NK cells, with helper T cells (CD4+) being more radiosensitive than cytotoxic T cells (CD8+). The preclinical studies support this difference between B and T cells. Preclinical studies also suggest that circulating lymphocytes appear to be more radiosensitive than non-circulating lymphocytes and tumor infiltrating T lymphocytes. In addition, parenchymal T cells from non-lymphoid solid organs appear to be more radioresistant than those from lymphoid solid organs. The obtained average dose-response alpha values derived from lymphocyte depletion in mice are ~0.8 Gy^-1^, ~0.6 Gy^-1^, and ~0.4 Gy^-1^, for B cells, T cells, and NK cells, respectively (for doses up to 3 Gy, after 4 hours to 4 days). For splenocytes, an average value of ~0.8 Gy^-1^ was extracted. There is some indication that naïve lymphocytes, which make up more than 50% of the lymphocyte population (depending on age, health status, and other factors) are more radiosensitive.

The reported *in vitro* data are less consistent than the *in vivo* results, but generally show the same ranking of radiosensitivity (B > T(CD8+) > T(CD4+) > NK) with response differences that are smaller than *in vivo*, i.e., average alpha values of ~0.4 Gy^-1^, ~0.3 Gy^-1^, and ~0.3 Gy^-1^ for B cells, T cells, and NK cells, respectively (for doses up to 3 Gy, after 4 hours to 4 days). One report shows significantly higher radiosensitivity for memory T cells. The fitted alpha values depend on the chosen dose range as most measured cell survival curves show a decreasing slope with increasing dose, i.e., a saturation typically starting already between 0.5 and 2 Gy. There is also a strong time dependence. Although radiation-induced apoptosis is measurable early after exposure, it continues to increase up to and beyond 48 hours, resulting in steeper dose-response curves. Many clinical studies on radiation-induced lymphopenia point out the importance of the dose to the circulating lymphocytes and refer to the high radiosensitivity of lymphocytes, often citing a single study (147). This widely cited study reports higher radiosensitivity than other studies and appears to be the only one showing a linear-quadratic dose response curve.

To assess the dose-response of lymphocytes *in vivo* for lymphopenia studies in patients, it is necessary to estimate the dose to lymphocytes. This work also reviews methods to estimate dose to the blood. While various models have been proposed to estimate the dose to the blood (based on reasonably well-known organ transit times of the blood), the dose to circulating lymphocytes is related but not identical to the blood dose. In addition to the recirculation of lymphocytes between blood and secondary lymphoid tissues, several factors cause lymphocyte transit times to be, on average, to be much longer than blood transit times. Lymphocytes often attach and detach from endothelial cells and radiation may cause upregulation of adhesion molecules that alter leucocyte adhesion to endothelial cells (171), which may increase mean transit times and thus dose to lymphocytes. In addition, they may have to deform to squeeze through capillaries because their size is much larger (~6 μm) than, for example, platelets (172, 173). In particular, pulmonary capillaries are thought to be slightly smaller than the diameter of lymphocytes (173). In the liver, a relatively low average velocity of T cells has been reported because they might be crawling on the endothelial wall of the sinusoids instead of flowing with the blood. This could reduce the average velocity to ~6-7 μm/min. Thus, CD8 T cells can super-diffuse in the liver for almost 20 minutes (172). It is therefore likely that the dose to the blood, while a potential surrogate for the dose to lymphocytes, does not accurately predict the correct dose experienced by circulating lymphocytes. Research efforts are underway to explicitly model lymphocyte trafficking rather than relying on the use of blood dose as a surrogate for dose to circulating lymphocytes (91, 92, 174).

While lymphocyte radiosensitivity is likely to play an important role in lymphopenia, radiation-induced effects such as cell survival or cell motility on lymphocytes are not necessarily robust predictors of immune suppression. Radiation also affects lymphocyte infiltration into tumors and tumor sensitization, increases antigen release, and other mechanisms (175). Zhao et al. (176) investigated lymphopenia in SBRT for early-stage lung cancer patients and concluded that lymphocyte radiosensitivity alone cannot explain lymphopenia without considering lymphocyte recovery times. The number of circulating lymphocytes might also decrease due to inflammation caused by low-dose baths in secondary irradiated organs during radiation therapy. In contrast to naïve adaptive lymphocytes which frequently migrate between secondary lymphoid organs, tissue-resident lymphocytes generally do not recirculate through the blood (177), but are also irradiated. The circulatory behavior of lymphocytes and their lymph node transit times also differs among lymphocytes subpopulations (178, 179), e.g., CD4+ seem to recirculate more rapidly compared to CD8+. In addition, radiation likely affects lymphocyte migration, for instance, through radiation-induced changes in sphingosine-1-phosphate (180). Lymphocyte depletion is also likely related to indirect mechanisms, such as radiation-induced expression of TNF-α which has a cytotoxic effect on lymphocytes (107, 181, 182). In addition, lymphocyte function appears to be affected at lower doses than cell survival in both *in vitro* and clinical studies (183–185).

## 5 Conclusions

The reported data suggest differences in radiation sensitivity among lymphocytes subpopulations, which may affect their relative contribution and thus the dynamics of the immune response. The data reviewed here show low dose (< 3Gy) radiosensitivity of lymphocytes in the same order of magnitude as normal fibroblasts (186). In general, B cells appear to be more radiosensitive than T cells, and NK cells appear to be the most resistant. Patient variability is likely to be of the same order of magnitude as the differences between subpopulations. Because tumor-infiltrating lymphocytes appear to be quite radioresistant, differences in radiosensitivity between circulating lymphocytes and lymphocytes in lymphoid organs may have implications for lymphopenia and thus for considerations of dose prescription and dose scheduling in radiation therapy as well as for fractionation and scheduling of therapies involving both radiation and immune checkpoint inhibitors. An important aspect is also the influence of radiation dose distribution, delivery time and beam arrangement, which has been discussed in the context of highly conformal radiotherapy and its positive effect on lymphopenia (114, 187).

It remains an open question whether the observed effects of radiation on lymphocyte counts in patients are indeed mainly due to the radiation sensitivity of circulating lymphocytes. To answer this question, it is necessary to consider not only the dose to different lymphocyte compartments in the field (e.g., the lymphatic system), but also radiation effects on lymphocyte trafficking and residence times (91, 92). Certainly, data on cell death don’t fully capture radiation-induced effects on lymphocyte functionality (135). This review outlines areas where additional research is needed to mechanistically explain radiation induced lymphopenia in patients and its correlation with treatment outcome.

## Author contributions

The author confirms being the sole contributor of this work and has approved it for publication.
